# Familial Non-Medullary Thyroid Carcinoma in Pediatric Age: Our Surgical Experience

**DOI:** 10.1007/s00268-021-06104-5

**Published:** 2021-04-23

**Authors:** Claudio Spinelli, Irene Piccolotti, Alessia Bertocchini, Riccardo Morganti, Gabriele Materazzi, Massimo Tonacchera, Silvia Strambi

**Affiliations:** 1 Pediatric and Adolescent Surgery Division, Department of Pathology Surgery, University of Pisa, Via Paradisa 2, 56124 Pisa, Italy; 2 Section of Statistics, Department of Clinical and Experimental Medicine, University of Pisa, Via Paradisa 2, 56124 PisaPisa, Italy; 3 Endocrine Surgery Division, Department of Surgical Pathology, University of Pisa, Via Paradisa 2, 56124 Pisa, Italy; 4 Endocrinology Unit, Department of Clinical and Experimental Medicine, University of Pisa, Via Paradisa 2, 56124 Pisa, Italy

## Abstract

**Background:**

The purpose of the article was to evaluate the existence of significant clinical, pathological and prognostic differences between familial and sporadic form of pediatric non-medullary thyroid carcinoma, in order to tailor the therapeutic strategy to be adopted for patients with family history.

**Methods:**

We analyzed the records of 76 pediatric patients that underwent surgery for differentiated thyroid cancer from 2014 to 2019 at the Surgical Pathology Department of the University of Pisa, Italy. Among these, 20 (26,3%) had positive family history (familial non-medullary thyroid carcinoma—FNMTC group) while 56 (73.7%) were affected by sporadic forms (sporadic non-medullary thyroid carcinoma—SNMTC group).

**Results:**

In our study, the correlation between the FNMTC and the SNMTC group showed no difference in terms of tumor features like multifocality, bilaterality, capsular/extracapsular invasion and the presence of vascular emboli. A statistical significance, on the other hand, was revealed by observation of clinical outcomes, such as distant metastasis (*p* = 0,022), persistence of disease (*p* = 0,054) and necessity of radioiodine sessions (*p* = 0,005).

**Conclusions:**

These findings suggest that family history may have an independent role on the outcome, expressing its action through an intrinsic more aggressive biological behavior. Therefore, familial non-medullary thyroid carcinoma in children represents a nosological entity that requires an accurate pre-operative evaluation, an adequate surgical strategy and a careful follow up.

## Introduction

Differentiated thyroid carcinoma accounts (DTC) for 0.5 to 3% of all childhood carcinomas and represents 90% of all thyroid cancers in childhood [1], while the medullary thyroid carcinoma (MTC) affects only 10% of pediatric cancer population [2]. Environmental risk factors for developing DTC are exposure to ionizing radiation, iodine deficiency, nodular and autoimmune disease and obesity [3–5]. With regard to genetic causes, the most frequently affected genes are RET / PTC, BRAF and RAS [6–8]. By now, pediatric thyroid carcinoma is considered a biologically different cancer from the adult one and there is a growing interest in identifying risk factors that can predict its aggressiveness and prognosis [9]. Family history is emerging among the risks and the negative prognostic factors, not only within the MEN form, of which the family and hereditary nature is widely known, but also in the differentiated, non-MEN, thyroid cancer [10]. This allows to divide differentiated thyroid carcinomas in two clinical entities: familial non-medullary thyroid carcinoma (FNMTC) and sporadic non-medullary thyroid carcinoma (SNMTC) [11]. According to the literature, familial cancers can account for about 10–15% of differentiated thyroid cancer of follicular derivation [12]. Hereditary forms of thyroid tumors have peculiar clinical and prognostic characteristics [13]. In fact, patients affected by FNMTC have a higher risk of bilaterality, multifocality, lymph node/remote invasion and recurrence [14], with a reduced disease-free survival [15]. Recognition of such patients would allow for more radical surgical treatment and accurate post-operative follow up [11]. Although family history has been recognized as a predictor of an unfavorable prognosis, there is still much to study on the weight of inheritance on outcome. Despite the increasing focus on this form of thyroid cancer, causative genes of FNMTC are still unknown; some studies suggest that the FNMTC has an autosomal dominant behavior with incomplete penetrance and variable expressivity [16, 17]. Due to the poor knowledge of the genetic basis of the FNMTC, a genetic test for FNMTC is not yet available [18, 19]. This study aims to analyze the weight of genetic inheritance on the tumor's anatomo-clinical features, on the surgical management and on the outcome of pediatric patients, in order to assess the importance of inheritance as an independent prognostic factor.

## Materials and methods

This is a retrospective study reviewing pediatric patients (≤18 years old) affected by Differentiated Thyroid Carcinoma (DTC), diagnosed and treated in the Pediatric and Adolescent Surgery Unit and in the Endocrine Surgery Unit of the University of Pisa (Italy) from January 2014 to December 2019. We included in the study 76 patients with a diagnosis of DTC confirmed on definitive histological examination by the Pathological Anatomy Unit of the same University. Collected records for each patients are family history of thyroid cancer, sex, age, pre-existing benign thyroid pathology (multinodular goiter and Hashimoto thyroiditis), clinical presentation, tumor size, surgery (thyroidectomy, hemithyroidectomy, lymphadenectomy of the central and lateral compartment, unilateral or bilateral), cervical lymph nodes metastases, distant metastases, multifocality, bilaterality, capsular invasion, extrathyroid invasion, neoplastic vascular emboli, histological examination, post-operative complications (recurrent laryngeal nerve palsy, transient hypocalcaemia, permanent hypocalcaemia), persistence or recurrence of disease, number of radiotherapy sessions received. Ultrasonography was performed in all patients before surgery, to determine tumor location, size and lymph node status; if the lesion had ultrasound features suggestive for thyroid cancer, we associated a fine needle aspiration biopsy (FNAB) of the mass. When a cervical lymph node was suspected of lymph node metastasis, we performed an additional FNAB to decide whether the patient needed therapeutic neck dissection or not. Thyroid function tests, including triiodiothyronine (T3), free T3 (FT3), thyroxine (T4), free T4 (FT4), thyroglobulin (Tg), thyroid-stimulating hormone (TSH), anti-peroxidase antibody (TPOAb) and anti-thyroglobulin antibody (TgAb), calcitonin and calcemia were performed before surgery. Other imaging studies, such as CT / RMN and radioactive iodine uptake test (RAIU test), were performed to evaluate lung metastases and mediastinal lymph nodes. The post-operative follow up was performed every 3 months for the first 2 years with clinical tests, ultrasound and blood tests (calcemia, TPOAb, TgAb, T3, FT3, T4, FT4, TSH and Tg levels) and then every 6 months. A chest x-ray or CT/RMN scan was obtained once a year. Patients were considered “disease-free” at follow up if they had a suppressed serum Tg < 1 ng/mL, no detectable TgAb, and no structural evidence of disease. Patients with Tg values ≥ 1 ng/mL, or stimulated Tg values ≥2 ng/mL, or any evidence of disease on imaging (ultrasonography or computed tomography scan, radioactive iodine scan) or biopsy-proven disease (cytology or histology) within one year from initial treatment were considered as having persistent disease. A recurrence was defined as new biochemical, structural, or functional evidence of disease that was detected after at least one year [20, 21].

Patients were divided into two groups based on the presence or absence of family history for thyroid cancer, emerging from the anamnestic data: Familial Non-Medullary Thyroid Carcinoma (FNMTC) and Sporadic Non-Medullary Thyroid Carcinoma (SNMTC). We included in the study all subjects with diagnosis of FNMTC (≤ 18 years old) with at least one first (parents) or second (siblings or uncles) degree relative diagnosed with differentiated thyroid carcinoma [22, 23].

## Data analysis

Categorical data were summarized by absolute and relative frequency, quantitative ones by mean or median and standard deviation or range. To compare the variable “central compartment” or “lateral compartment” with the qualitative risk factors, chi-square test and z-test for two proportions were used, while for the quantitative risk factors Student's t test for independent samples (two tailed) was applied. Significance was set at 0.05. All analyzes were performed with SPSS version 26 technology (Chicago, Illinois, USA).

## Results

The average age of onset of DTC in our 76 patients was 15.14 years (range 8–18). The average age in the FNMTC group was 15.45 years (range 10–18), while in the SNMTC group was 14.99 years (range 8–18) (p = 0,286). The patients consisted of 22 males (29%) and 54 females (71%). Among the FNMTC subjects there were 14 female (70%) and 6 males (30%); in the SNMTC sample, females were 40 (71.4%) and males 16 (28.6%) (p = 0,868). Of the 76 patients examined, 20 patients (26.3%) belonged to the FNMTC group. Of these patients, 5 (6.6% of the total) had first-degree inheritance for thyroid cancer, while 15 (19.7% of the total) had second-degree inheritance. Of the 20 patients with hereditary thyroid cancer, 13 (65%) were familial along the maternal line, 6 (30%) from the paternal line, while one (5%) patient had a positive family history of carcinoma on both inherit lines. This comparison was statistically significant, with p < 0,001. Pre-existing benign thyroid pathology was found in 5 (25%) patients in the FNMTC group, while in 9 (16%) in the SNMTC one (p = 0,584). No significant differences were found between the two groups as regards the parameters: clinical presentation, tumor size, surgery, lymph nodes metastases, multifocality, bilaterality, capsule invasion, extrathyroidal invasion, neoplastic embolism and recurrences. Regarding histological examination, classical variant of papillary carcinoma was present in 15 cases (75%) of FNMTC and in 36 cases (64.3%) of SNMTC (p = 0,550) while follicular variant in 2 cases (10%) of FNMTC and in 10 cases (17.85%) of the SNMTC one; the analysis of histological subtypes did not reveal a significant difference between the two groups (Tables 1, 2). Regarding outcomes, interesting statistically significant differences emerged for distant metastasis (all located at the lung level), persistence of disease and number of radioiodine (RAI) sessions. Distant metastases were found in 3 (15%) familial subjects, while were absent in the patients affected by sporadic carcinoma (*p* = 0,022). Persistence of disease occurred in 6 (30%) patients of the FNMTC group and in 4 (7.1%) of SNMTC group (*p* = 0,054). The difference between the average number of RAI sessions for patient in the two groups is statistically significant, with *p* = 0,005 (Table 3).

**Table 1:** Comparison of the clinical factors between the FNMTC group and the SNMTC group

	FNMTC (20)	SNMTC (56)	p value
*Familiarity*			
Maternal line	13 (65%)		
Paternal line	6 (30%)		
Both (Maternal-Paternal line)	1 (5%)		
*Sex*			0.868
Male	6 (30%)	16 (28.6%)	
Female	14 (70%)	40 (71.4%)	
*Age*			0.286
Average	15.45 (± 1.8)	14.99 (± 1.6)	
*Previous benign thyroid disease*			0.584
Yes	5 (25%)	9 (16.1%)	
No	15 (75%)	47 (83.9%)	
*Clinical presentation*			0.461
Thyroid nodule	15 (75%)	35 (62.5%)	
Thyroid nodule + limphadenopathy	5 (25%)	21 (37.5%)	
*Tumor size*			0.967
< 2 cm	12 (60%)	32 (57.1%)	
> 2 cm	8 (40%)	24 (42.9%)

**Table 2:** Comparison of the surgical and anatomo-pathological factors between the FNMTC group and the SNMTC group. Statistics: frequency (%) or mean (± sd)

Factor	FNMTC (20)	SNMTC (56)	p value
*Surgery*			0.758
Lobectomy	1 (5%)	6 (10.7%)	
**Thyroidectomy (Tx)**	**19 (95%)**	**50 (89.3%)**	
**Lymphadenectomy (CC or LC)**			0.392
No lymphadenectomy	10 (50%)	36 (64.3%)	0.392
Yes lymphadenectomy (L)	10 (50%)	20 (35.7%)	
L-Tx + cc unilateral	6 (30%)	4 (7.1%)	0.264
L-Tx + lc unilateral	0 (0%)	1 (1.8%)	0.175
L-Tx + cc + lc unilateral	3 (15%)	14 (25%)	0.875
L-Tx + cc + lc bilateral	1 (5%)	1 (1.8%)	0.506
*Metastatic Cervical Compartments (N +)*			0.654
No lymphonodal metastases	4 (20%)	7 (12.5%)	
Yes Lymphonodal metastases (LM)	16 (80%)	49 (87.5%)	
LM-Central compartment (CC)	8 (40%)	19 (33.9%)	0.887
LM-Lateral compartment (LC)	4 (20%)	15 (26.8%)	0.832
LM-Both central and lateral compartments (CC + LC)	3 (15%)	14 (25%)	0.627
LM-CC + LC bilateraly	1 (5%)	1 (1.8%)	0.838
*Lymph nodes removed*			
Average lymph nodes removed (N) / patient	12.2 (± 2.8)	15.13 (± 3.7)	**0.002**
Average metastatic lymph nodes (N +) / patient	4 (± 2.6)	5.05 (± 2.7)	**0.038**
Removed/metastatic lymph nodes rate	0.327 (± 0.1)	0.333 (± 0.1)	0.820
*Multifocality*			0.967
Yes	8 (40%)	24 (42.9%)	
No	12 (60%)	32 (57.1%)	
*Bilaterality*			0.901
Yes	3 (15%)	11 (19.6%)	
No	17 (85%)	45 (80.4%)	
*Capsular invasion*			0.650
Yes	17 (85%)	43 (76.8%)	
No	3 (15%)	13 (23.2%)	
*Extrathyroid invasion*			0.181
Yes	7 (35%)	20 (35.7%)	
No	13 (65%)	36 (64.3%)	
*Neoplastic vascular emboli*			0.519
None	8 (40%)	29 (51.8%)	
Yes (Y)	12 (60%)	27 (48.2%)	
Y > 4	5 (25%)	17 (30.3%)	0.375
Y < 4	7 (35%)	10 (17.9%)	-
*Histology*			
Follicular Carcinoma	0 (0%)	0 (0%)	> 0.05
Papillar Carcinoma (PC)	20 (100%)	56 (100%)	-
PC-Classical Variant	15 (75%)	36 (64.3%)	0.550
PC-Follicular variant	2 (10%)	10 (17.9%)	0.642
PC-Solid	1 (5%)	5 (8.8%)	0.939
PC-High cell	1 (5%)	3 (5.4%)	0.602
PC-Diffuse sclerosing variant	1 (5%)	2 (3.6%)	0.699

**Table 3:**
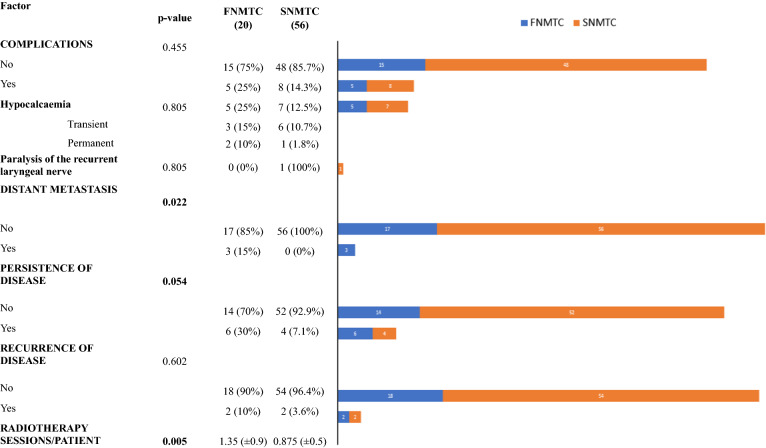
Comparison of the post-operative complications and prognostic factors between the FNMTC group and the SNMTC group. Statistics: frequency (%) or mean (± sd)

## Discussion and conclusions

The present study compares a group of pediatric subjects with Familial Non-Medullary Thyroid Carcinoma with a control group consisting of pediatric subjects affected by a sporadic form.

Regarding the clinicopathological features, in our casuistry, there were no significant differences between the two groups. We registered a prevalence of pre-existing benign thyroid pathology in familial forms of 25%, which is lower than data reported in the literature [18, 24]; for example, Haggie Mazeh et al. [13] described a prevalence of pre-existing benign thyroid disease of 45%.

According to the literature [25–27], the clinical presentation of the tumor as well as its size at diagnosis was equally distributed in the two groups (*p* = 0,967). Regarding surgical treatment, no major use of thyroidectomy was found in subjects with familial disease (95%) compared to sporadic forms (89%) (*p* = 0.758). Lymphadenectomy was performed on half of the familial subjects and on 35% of the sporadic cases. Regarding lymph node metastases, there were no significant differences in the two groups, and this was also confirmed by the multivariate statistical analysis. Similar results were found for the number of lymph nodes removed, the number of metastatic lymph nodes and their relationship. In our study, there was no difference in the analysis of multifocality, bilaterality, capsular and extracapsular invasion and presence of vascular emboli, and these results are in conflict with other studies we found in the literature [14, 15] that reported a more aggressive local presentation for familial carcinomas. Uchino et al. [14] stated that, compared to the patients with sporadic disease, the FNMTC patients were more likely to have multifocal disease (40.7% for FNMTC vs. 28.5% for SNMTC, *p* < 0.0001), while in our study, the prevalence of multifocality was similar in the two groups (40% for FNMTC vs. 42.9% for SNMTC, *p* = 0.967).

Interesting data, on the other hand, emerged in our study from the analysis of clinical outcomes, such as complications, persistence and recurrence of disease, necessary radioiodine sessions and distant metastasis. In the literature, there is a lack of studies on post-operative complications of thyroidectomy in the pediatric population and in particular of specific studies on the family form of differentiated thyroid carcinoma. In our study, FNMTC group had a slightly higher post-operative complication rate but this difference was not statistically significant (*p* = 0.455). Incidence of distance metastases in FNMTC group in our casuistry was 15% higher compared to control group, with a statistically significant difference (*p* value = 0,022). Robenshtok et al. [28] reported a higher incidence (8%) in patients with a familial form than in those with a sporadic form (5%), while other authors [29, 30] have found no increase in distant metastasis in familial form.

In our study, the incidence of persistence of disease in patients with FNMTC was significantly higher than in patients affected by SNMTC (*p* = 0,054) and this finding is in line with recent literature [13, 29, 31–33]. Other interesting data concern the average number of necessary radiotherapy sessions in the two groups, with a statistically significant difference (*p* value = 0.005). From another point of view, as highlighted by Hillenbrand A. et al. [34], a greater number of radiotherapy sessions for FNMTC confirms a reduction in disease-free survival in these subjects, in accordance with the increase in distant metastases and persistence of disease. The discrepancy between these data and those that emerged from the analysis of local invasiveness features suggests that inheritance is an independent negative prognostic factor with respect to the local invasiveness of the tumor, which was comparable to the sporadic forms and this is probably due to a different genetic biology of the tumor [35]. Although the local extent of the tumor has not been enhanced by inheritance, the forms of FNMTC presented a worse outcome in terms of risk of distant metastasis, persistence of disease and average number of radiotherapy sessions for patient. These findings suggest that family history may have an independent role on the outcome, expressing its action through an intrinsic more aggressive biological behavior. Therefore, familial non-medullary thyroid carcinoma in children represents a nosological entity that requires an accurate pre-operative evaluation, an adequate surgical strategy and a careful follow-up.

The study is limited by its retrospective and registry-based nature, and thus some data can be missing. In addition, since the familiarity was found from the anamnesis collected by the patient’s parents, often without any personal medical documentation, some distortions due to recall mistakes in the family history can have been included. Nevertheless, we hope that our study can pave the way for other research on the familial form of differentiated thyroid carcinoma in pediatric age.
